# Synergistic Induction of Caspase-8-Mediated Leukaemic Cell Death by Fisetin and Pinocembrin

**DOI:** 10.3390/ijms27125622

**Published:** 2026-06-22

**Authors:** Narawan Kaewthawee, Ankita Sharma, James Michael Brimson, Sirikalaya Brimson

**Affiliations:** 1 Program in Clinical Hematology Sciences, Department of Clinical Microscopy, Faculty of Allied Health Sciences, Chulalongkorn University, Bangkok 10330, Thailand; 6271006137@student.chula.ac.th; 2 Research Innovation & International Affairs, Faculty of Allied Health Sciences, Chulalongkorn University, Bangkok 10330, Thailand; ankita.s@chula.ac.th (A.S.); jamesmichael.b@chula.ac.th (J.M.B.); 3 Department of Clinical Microscopy, Faculty of Allied Health Sciences, Chulalongkorn University, Bangkok 10330, Thailand

**Keywords:** death-inducing signalling complex (DISC), FADD adaptor protein, TRAIL receptors (DR4/DR5), autophagy flux, cell cycle arrest, combination therapy, chemotherapy sensitisation

## Abstract

Fisetin is a bioactive flavanol with reported anticancer activity, although its mechanisms in leukaemia and potential for combination therapy remain incompletely understood. This study investigated the cytotoxic and mechanistic effects of fisetin, alone and combined with pinocembrin, in human leukaemia cells. Cell viability, apoptosis, and cell cycle progression were assessed by flow cytometry; protein expression in Jurkat cells was assessed by Western blotting; and molecular docking was used to evaluate interactions with the Fas receptor. Drug interactions were quantified using ZIP synergy analysis, and cytotoxicity and clonogenic survival were evaluated using soft-agar colony formation assays in K562 cells. Fisetin significantly reduced cell viability and induced apoptosis, accompanied by caspase-8 cleavage, p62 accumulation, and CDK4 downregulation, consistent with activation of extrinsic apoptosis, impaired autophagic flux, and cell cycle inhibition in Jurkat cells. Docking analysis supported a potential interaction with the Fas receptor, which was confirmed using the Fas receptor antagonist Met-12. Co-treatment with pinocembrin enhanced fisetin-mediated cytotoxicity and produced synergistic effects, particularly in Jurkat cells (ZIP score > 10), while synergistic interactions at specific sub-IC_50_ concentrations were also observed in K562 cells. Combination treatment further enhanced caspase-8 activation, reduced CDK4 expression in Jurkat cells, and significantly suppressed clonogenic survival in K562 cells compared with single-agent treatments. These findings suggest that fisetin promotes caspase-8-dependent apoptosis, potentially involving Fas-associated signalling, and highlight fisetin–pinocembrin combination therapy as a promising strategy for leukaemia treatment.

## 1. Introduction

Acute lymphoblastic leukaemia (ALL) is a type of haematologic malignancy characterised by the uncontrolled proliferation of abnormal lymphoid progenitor cells, which expand and replace normal haematopoietic cells in the bone marrow and spread to the peripheral blood and various extramedullary organs. Patients with ALL may present with nonspecific signs and symptoms, such as anaemia, fever, infections, and easy bleeding or bruising due to low blood cell counts and impaired function [1,2]. Standard treatment options for acute lymphoblastic leukaemia include chemotherapy, radiotherapy, allogeneic haematopoietic stem cell transplantation, targeted therapy and immunotherapy, including CAR-T cell therapy. However, these treatments are frequently associated with significant adverse effects [3,4,5]. Consequently, there is growing interest in alternative and complementary therapies, particularly plant-derived phytochemicals [6,7], which are being investigated both as complementary treatments to conventional therapies to reduce the side effects and as potential novel therapeutic agents.

**Figure 1:**
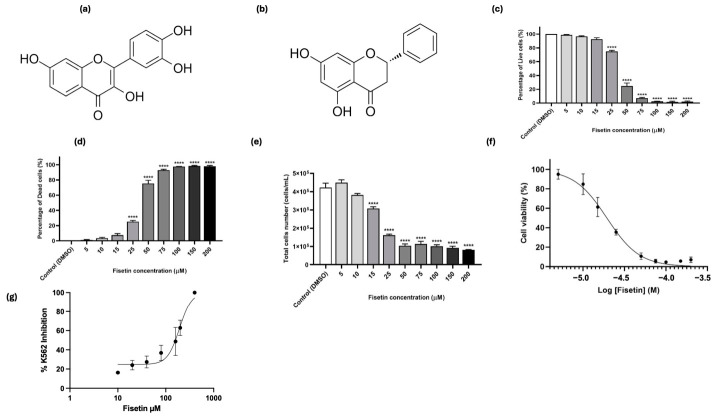
Chemical structures of (**a**) fisetin and (**b**) pinocembrin. The effect of fisetin on Jurkat cell viability was evaluated using the trypan blue exclusion assay. The percentage of live cells (**c**) and dead cells (**d**), as well as the total cell number (**e**), was determined after treatment with fisetin for 48 h. In addition, (**f**) cell viability in Jurkat and (**g**) the effect of fisetin on K562 cell viability were assessed by the XTT assay following treatment of cells with various concentrations of fisetin for 48 h. The half-maximal inhibitory concentration (IC_50_) of fisetin is 19.23 ± 1.14 µM in Jurkat cells and 193.3 ± 1.14 µM in K562 cells. Statistical significance was analysed using one-way ANOVA followed by Tukey’s *post hoc* test compared with the control group (**** *p* < 0.0001) (*n* = 3).

Fisetin (3,3′,4′,7-tetrahydroxyflavone) is a bioactive, chemically defined flavanol (Figure 1a) found in a variety of plants, vegetables, herbs, and fruits, with strawberries being the richest source [8,9,10]. Fisetin exhibits diverse pharmacological activities, most notably its anticancer effects. It inhibits key signalling pathways and regulates multiple proteins involved in cancer cell growth, survival, and proliferation. Fisetin acts at various stages of tumour development by inhibiting carcinogenesis, proliferation, and angiogenesis, and by inducing cell cycle arrest, apoptosis, and autophagy [11,12,13,14,15,16]. However, whether fisetin exerts consistent anti-leukaemic effects across distinct haematological malignancies and whether it can enhance therapeutic responses in combination strategies remain to be fully elucidated.

Autophagy and apoptosis are interconnected cellular processes that regulate cell survival and death. While autophagy generally supports cell viability, its dysregulation can lead to cell death [17,18]. Apoptosis, or programmed cell death, occurs through two main signalling pathways: the intrinsic (mitochondrial) pathway and the extrinsic (death receptor) pathway [19,20]. The intrinsic pathway is triggered by intracellular stress signals and regulated by Bcl-2 family proteins. It involves the release of cytochrome c (Cyt c) and apoptosis-inducing factor (AIF) from mitochondria. Cytochrome c then binds to apoptotic protease-activating factor-1 (APAF-1), leading to formation of the apoptosome and activation of downstream signalling events. In contrast, the extrinsic pathway is initiated when extracellular ligands bind to cell-surface death receptors, such as the Fas receptor (CD95/Apo-1). This binding recruits the Fas-associated death domain (FADD) and activates caspase-8 via the death-inducing signalling complex (DISC). Both pathways ultimately activate caspase-3, resulting in DNA fragmentation, apoptotic cell death, and subsequent phagocytic clearance [21,22,23].

Fisetin has been reported to exhibit significant anticancer activity against various cancer types. In breast cancer (MCF-7 cells), it inhibits invasion by suppressing MMP-9 activation via the PKC/ROS/MAPK pathways [24]. In lung cancer (A549 cells), it suppresses migration and invasion by attenuating epithelial-to-mesenchymal transition (EMT) [25]. In liver cancer (HepG2 cells), it inhibits autophagy via the PI3K/Akt/mTOR and AMPK pathways [26]. In HeLa cells (a cervical cancer cell line), fisetin inhibits cell proliferation, enhances apoptosis, reduces oxidative stress, and alleviates inflammation by regulating the JAK-STAT/NF-κB pathways [27]. In multiple myeloma (U266 cells), it stimulates reactive oxygen species (ROS) production, leading to apoptosis signalling, and activates AMP-activated protein kinase (AMPK) signalling [28]. In Burkitt’s lymphoma (Raji cells), it induces apoptotic cell death, suppresses the mammalian target of rapamycin (mTOR) pathway, and modulates phosphatidylinositol 3-kinase (PI3K) signalling [29]. In haematological malignancies, fisetin dose-dependently inhibits cell proliferation, induces apoptosis by increasing caspase-3 activation, and causes S- and G2/M-phase cell cycle arrest via the JAK/STAT signalling pathway in chronic myeloid leukaemia (K562 cells) [30]. In acute promyelocytic leukaemia (HL60 cells), it inhibits proliferation, induces G2/M-phase cell cycle arrest, enhances caspase-3 activity, and modulates the MAPK pathway [31]. Although fisetin has shown promising anticancer activity, its mechanistic effects in leukaemic T cells, its impact on long-term proliferative capacity, and its broader applicability across leukaemia subtypes remain incompletely understood. In addition, combination strategies involving bioactive compounds represent a promising strategy to enhance therapeutic efficacy while minimising toxicity associated with high-dose chemotherapy. Pinocembrin, a naturally occurring flavonoid, has previously been reported to exhibit anticancer activity and induce G0/G1 cell cycle arrest through CDK4 regulation in leukaemic cells [32]. However, whether pinocembrin can modulate and enhance fisetin-mediated anti-leukaemic activity has not been investigated. Therefore, the potential of fisetin to enhance therapeutic efficacy in combination strategies requires further investigation. So, this study aims to investigate the effects of fisetin, alone or in combination with other compounds, in leukaemic cellular models, with a particular focus on its role in regulating cell proliferation and apoptotic signalling pathways in Jurkat cells while further evaluating cytotoxic and synergistic effects in K562 chronic myelogenous leukaemia cells along with clonogenic assays to assess the long-term impact of treatment on leukaemic cell survival.

## 2. Results

### 2.1. Fisetin-Mediated Effects on Cell Viability

The chemical structures of fisetin and pinocembrin are shown in Figure 1a and Figure 1b, respectively. Jurkat cells were treated with fisetin at concentrations of 5 to 200 µM for 48 h. After treatment, cells were stained with 0.4% trypan blue. In Figure 1c, the percentage of live cells decreased significantly with increasing fisetin concentration. Notably, at concentrations of 25 µM and above, cell viability remained low (*p* < 0.0001). Similarly, in Figure 1e, the total cell number decreased significantly following fisetin treatment. In addition, the percentage of dead cells increased significantly in a concentration-dependent manner (Figure 1d), indicating inhibited cell proliferation, increased cytotoxicity and induction of cell death by fisetin.

**Figure 2:**
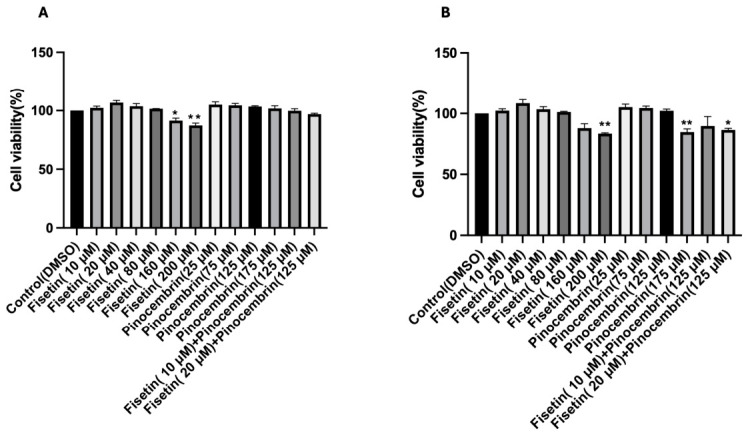
Fisetin and pinocembrin toxicity measured using the MTT assay. (**A**) HaCaT cells and (**B**) HEK 293 cells. Statistical significance calculated using ANOVA, followed by Dunnett’s *post hoc* test—compared to control (* *p* < 0.05; ** *p* < 0.01) (*n* = 3).

To compare the cytotoxic effects of fisetin and pinocembrin across different leukaemia models, cell viability was assessed in both Jurkat and K562 cells using the XTT assay following treatment with increasing concentrations of each compound for 48 h, which showed a concentration-dependent reduction in cell viability compared with the control (DMSO < 1%) in both cell lines. The half-maximal inhibitory concentration (IC_50_) for fisetin in Jurkat was 19.23 ± 1.14 µM (Figure 1f), and in K562 was 193.7 µM, indicating reduced sensitivity of K562 cells to fisetin (Figure 1g). In contrast, no significant cytotoxicity was observed in HEK293 and HaCaT cells under identical conditions (Figure 2).

### 2.2. Fisetin-Mediated Effects on Cell Cycle in Jurkat Cells

**Figure 3:**
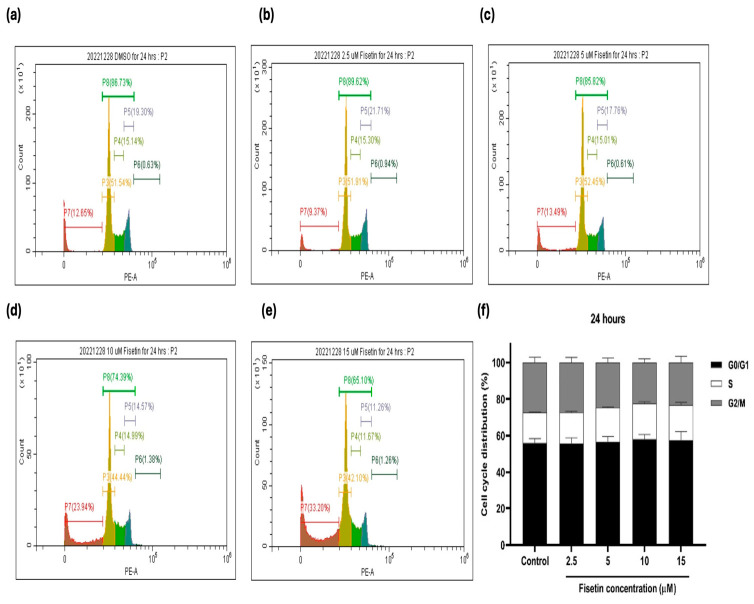
Cell cycle analysis of Jurkat cells following fisetin treatment as determined by propidium iodide staining. (**a**–**e**) Representative figures for Jurkat cells were treated with fisetin at concentrations ranging from 2.5 to 15 µM for 24 h. Cells were subsequently stained with a propidium iodide/RNase solution and analysed by flow cytometry. (**f**) The average percentages of cells in the G0/G1, S, and G2/M phases obtained from three independent experiments (*n* = 3).

**Figure 4:**
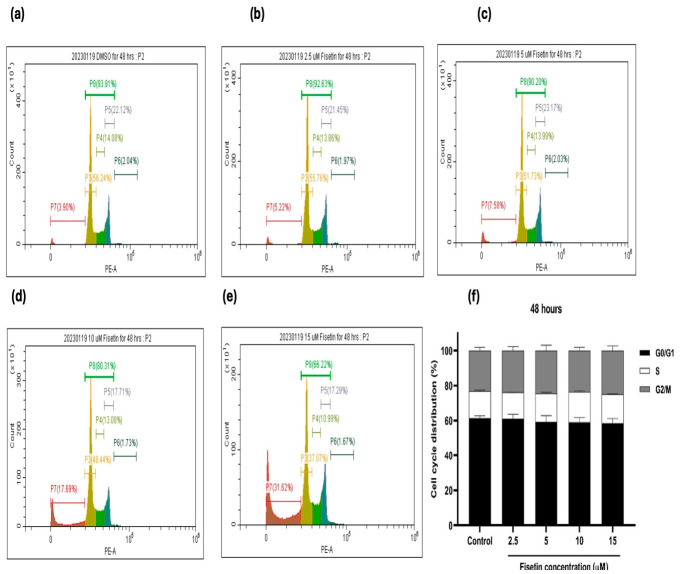
Cell cycle analysis of Jurkat cells following fisetin treatment as determined by propidium iodide staining. (**a**–**e**) Jurkat cells were treated with fisetin at concentrations ranging from 2.5 to 15 µM for 48 h. Cells were subsequently stained with a propidium iodide/RNase solution and analysed by flow cytometry. (**f**) The average percentages of cells in the G0/G1, S, and G2/M phases obtained from three independent experiments (*n* = 3).

**Figure 5:**
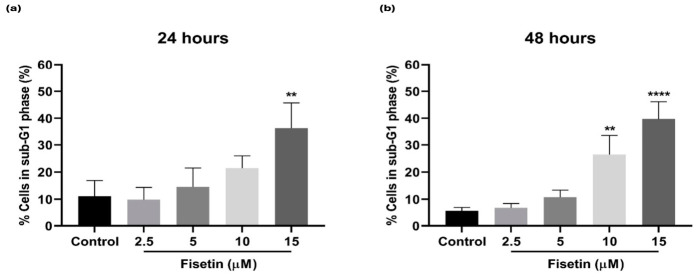
Flow cytometric analysis of cell cycle distribution focusing on the sub-G1 fraction in Jurkat cells after fisetin treatment. Jurkat cells were treated with fisetin at concentrations ranging from 2.5 to 15 µM for (**a**) 24 h and (**b**) 48 h. The data are presented as the mean percentage ± SEM of cells in the sub-G1 population relative to the total cell count, obtained from three independent experiments (*n* = 3). Statistical significance was analysed using one-way ANOVA followed by Tukey’s *post hoc* test compared with the control group. (** *p* < 0.01, **** *p* < 0.0001).

To assess the effect of fisetin on cell cycle progression, cells were stained with propidium iodide and RNase, and then analysed by flow cytometry. Jurkat cells were treated with fisetin at concentrations of 2.5, 5, 10 and 15 µM for 24 and 48 h. The results showed that fisetin did not cause statistically significant changes in the distribution of cells across the G0/G1, S and G2/M phases at 24 h (Figure 3a–e) and 48 h (Figure 4a–e) compared with the control (DMSO < 1%). However, a concentration-dependent increase in the sub-G1 population was observed. As shown in Figure 4, flow cytometry analysis demonstrated that fisetin significantly increased the number of apoptotic cells in both a time- and concentration-dependent manner, as evidenced by increased accumulation of cells in the sub-G1 phase at 24 h (Figure 5a) and 48 h (Figure 5b), indicating the induction of apoptosis. These findings suggest that fisetin induces apoptotic cell death rather than causing a distinct cell cycle arrest in Jurkat cells.

### 2.3. Fisetin-Mediated Effects on Apoptosis Activation in Jurkat Cells

**Figure 6:**
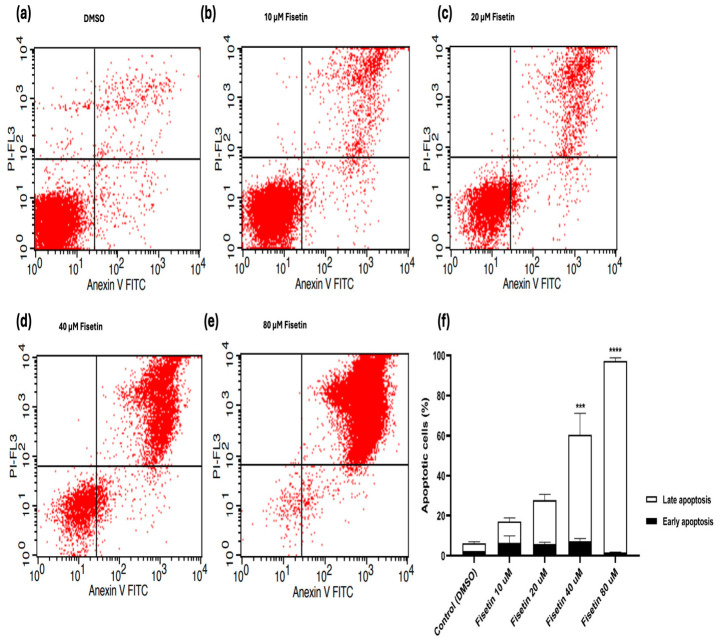
(**a**–**e**) Representative flow cytometry analyses showing Annexin V/propidium iodide (PI) staining of Jurkat cells after fisetin treatment. Jurkat cells were treated with fisetin at concentrations ranging from 10 to 80 µM for 48 h and subsequently stained with Annexin V and PI to evaluate apoptotic cell populations. (**f**) Data are presented as the mean percentage ± SEM of apoptotic cells from three independent experiments (*n* = 3). Statistical significance was determined using one-way ANOVA followed by Tukey’s *post hoc* test compared with the control group (*** *p* < 0.001, **** *p* < 0.0001).

Building on previous findings that fisetin induces apoptotic cell death, we treated Jurkat cells with fisetin at concentrations of 10, 20, 40, and 80 μM for 48 h and assessed apoptosis using Annexin V/PI staining. The results showed that fisetin significantly increased the percentage of apoptotic cells in a concentration-dependent manner compared with the control (DMSO < 1%), as shown in Figure 6a–f. The proportion of apoptotic cells increased from 6.18 ± 0.71% in the control group to 16.97 ± 4.94%, 27.88 ± 3.02%, 60.50 ± 9.43%, and 97.36 ± 1.34% at 10, 20, 40, and 80 μM fisetin, respectively. Notably, a marked increase in apoptotic cells was observed at higher concentrations (40 and 80 μM). These findings indicate that fisetin effectively induces apoptosis in Jurkat cells in a concentration-dependent manner.

### 2.4. Fisetin-Mediated Effects on Apoptotic Protein Expression in Jurkat Cells

**Figure 7:**
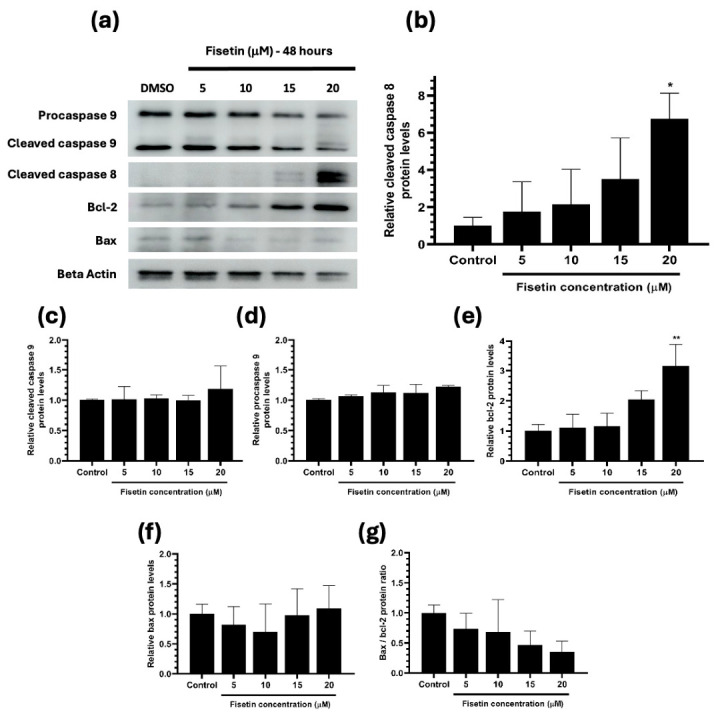
(**a**–**g**) Effect of fisetin on apoptosis-related protein expression in Jurkat cells. Jurkat cells were treated with fisetin at concentrations ranging from 5 to 20 µM for 48 h. Protein expression levels were determined by Western blot analysis. Relative protein expression levels are presented as the mean ± SEM from three independent experiments (*n* = 3). Statistical significance was determined using one-way ANOVA followed by Tukey’s *post hoc* test compared with the control group (* *p* < 0.05, ** *p* < 0.01).

As previously described, fisetin induces apoptotic cell death, as evidenced by reduced cell proliferation and increased apoptotic cell populations. Therefore, we evaluated the effect of fisetin on apoptosis-related proteins in Jurkat cells (Figure 7a–g). The cells were treated with fisetin at concentrations of 5, 10, 15 and 20 µM for 48 h, and protein expression levels were determined by Western blot analysis. The results showed that fisetin significantly increased cleaved caspase-8 protein expression in a concentration-dependent manner, particularly at higher concentrations (20 µM), as shown in Figure 6b, indicating activation of the extrinsic apoptosis pathway.

In contrast, cleaved caspase-9 protein expression showed no statistically significant changes across the various concentrations of fisetin (Figure 7c), while procaspase-9 levels remained relatively constant (Figure 7d). However, for Bcl-2 family proteins, fisetin treatment increased Bcl-2 (anti-apoptotic protein) expression, which was statistically significant at 20 µM (Figure 7e). In contrast, Bax (pro-apoptotic protein) expression decreased slightly with increasing fisetin concentrations (Figure 6f), resulting in a reduced Bax/Bcl-2 ratio (Figure 7g). However, this change was not statistically significant. These findings further support the view that fisetin may not effectively activate the mitochondrial (intrinsic) apoptotic pathway under these conditions. These results suggest that fisetin induces apoptosis in Jurkat cells, potentially through activation of the extrinsic apoptotic pathway, as indicated by increased caspase-8 activation, rather than the intrinsic mitochondrial pathway. However, although the observed increase in Bcl-2 expression may be attributable to alternative mechanisms, such findings are commonly reported in drug-resistant cancers and may be associated with tumour progression or enhanced cellular survival, particularly through autophagy regulation [33].

### 2.5. Fisetin-Mediated Effects on Protein Expression Related to Autophagy in Jurkat Cells

**Figure 8:**
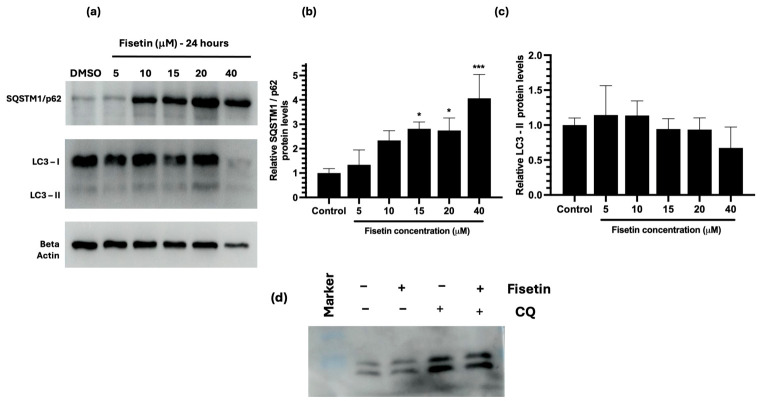
Effect of fisetin on autophagy-related protein expression in Jurkat cells. Jurkat cells were treated with fisetin at concentrations ranging from 5 to 40 µM for 24 h. (**a**) Protein expression levels were determined by Western blot analysis. (**b**,**c**) Relative protein expression levels are presented as the mean ± SEM from three independent experiments (*n* = 3). (**d**) Fisetin (20µM) effect on LC3B expression in the presence and absence of chloroquine (CQ) (20 µM). Statistical significance was determined using one-way ANOVA followed by Tukey’s *post hoc* test compared with the control group (* *p* < 0.05, *** *p* < 0.001).

The autophagy mechanism is initiated as an early adaptive response to cellular stress and functions as a pro-survival process that occurs before apoptosis [18]. Therefore, we evaluated the effect of fisetin on the expression of autophagy-related proteins in Jurkat cells over a 24 h period. Jurkat cells were treated with fisetin at concentrations of 5, 10, 15, 20 and 40 µM for 24 h, and protein expression levels were determined by Western blot analysis (Figure 8a–c). The results showed that SQSTM1/p62 expression increased in a concentration-dependent manner following fisetin treatment. As shown in Figure 7b, there was a significant increase in p62 protein levels at concentrations of 15, 20 and 40 µM, with the highest level observed at 40 µM (*p* < 0.001).

In contrast, LC3 protein levels showed no statistically significant changes across the various fisetin concentrations (Figure 8c). Although slight fluctuations were observed, LC3 expression tended to decrease at higher concentrations, particularly at 40 µM, suggesting suppressed autophagic signalling/homeostasis. Furthermore, no significant change in LC3B was observed in the presence and absence of fisetin, and when fisetin was added with and without chloroquine (Figure 8d). 

Collectively, the findings indicate that fisetin alters autophagy-related signalling, as evidenced by accumulation of SQSTM1/p62 and changes in LC3B expression. While these findings are consistent with disruption of autophagy homeostasis, additional studies using dedicated flux assays are required to definitively determine the effect of fisetin on autophagic flux.

### 2.6. Molecular Docking Analysis of Fisetin Binding to the Fas Receptor

**Figure 9:**
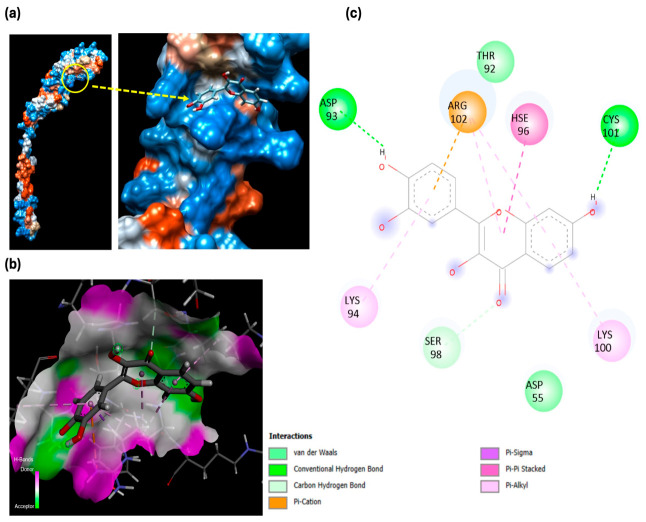
Molecular docking analysis (SwissDock docking platform) of fisetin binding to the Fas receptor (AlphaFold Protein Structure Database (AlphaFold ID: AF-P25445-F1-v6)). (**a**) The 3D surface representation of the predicted binding mode of fisetin within the Fas receptor binding pocket. The surface is coloured according to electrostatic potential: blue indicates positively charged regions, red/orange indicates negatively charged regions, and white/neutral indicates neutral or hydrophobic regions. (**b**) The 3D and (**c**) 2D interaction diagrams of fisetin docked into the Fas receptor, visualised using BIOVIA Discovery Studio v25.1.0.24284. Conventional hydrogen bonds (green dashed lines) are formed between the ligand and ASP93 as well as CYS101, contributing to the stability of the ligand–receptor complex. In addition, a π–cation interaction (orange dashed line) is observed with ARG102, while π–alkyl interactions (light pink dashed lines) occur with LYS94 and LYS100.

Previously, fisetin significantly increased cleaved caspase-8 expression in a concentration-dependent manner, indicating activation of the extrinsic apoptotic pathway. This pathway is initiated by the Fas receptor, a cell-surface receptor that binds extracellular ligands, thereby activating caspase-8. To further investigate the underlying molecular mechanism, molecular docking analysis was conducted using the Fas receptor as the target protein and fisetin as the ligand. According to this model, fisetin exhibited a binding free energy (ΔG) of −7.0274 kcal/mol when interacting with the Fas receptor. The docking analysis showed that fisetin interacts with the Fas receptor through multiple non-covalent interactions (Figure 9a–c). The ligand formed conventional hydrogen bonds (green dashed lines) with residues Asp93 and Cys101, contributing to the stability of the ligand–receptor complex.

Additionally, a carbon–hydrogen bond was observed at Ser98. A π–cation interaction (orange dashed line) was identified between fisetin and Arg102, indicating a strong electrostatic contribution to ligand binding. Furthermore, several π-related interactions, including π-π stacking and π–alkyl interactions (pink dashed line), were detected involving residues such as Hse96, Lys94, and Lys100, thereby enhancing hydrophobic stabilisation. Other residues in the vicinity, including Thr92 and Asp55, engaged in van der Waals interactions, further supporting fisetin’s binding affinity within the Fas receptor’s active site. These results indicate that fisetin-induced apoptosis in leukaemic T cells may be mediated through the Fas-dependent extrinsic apoptotic pathway.

### 2.7. Fisetin Effect on Fas Receptor Signalling

**Figure 10:**
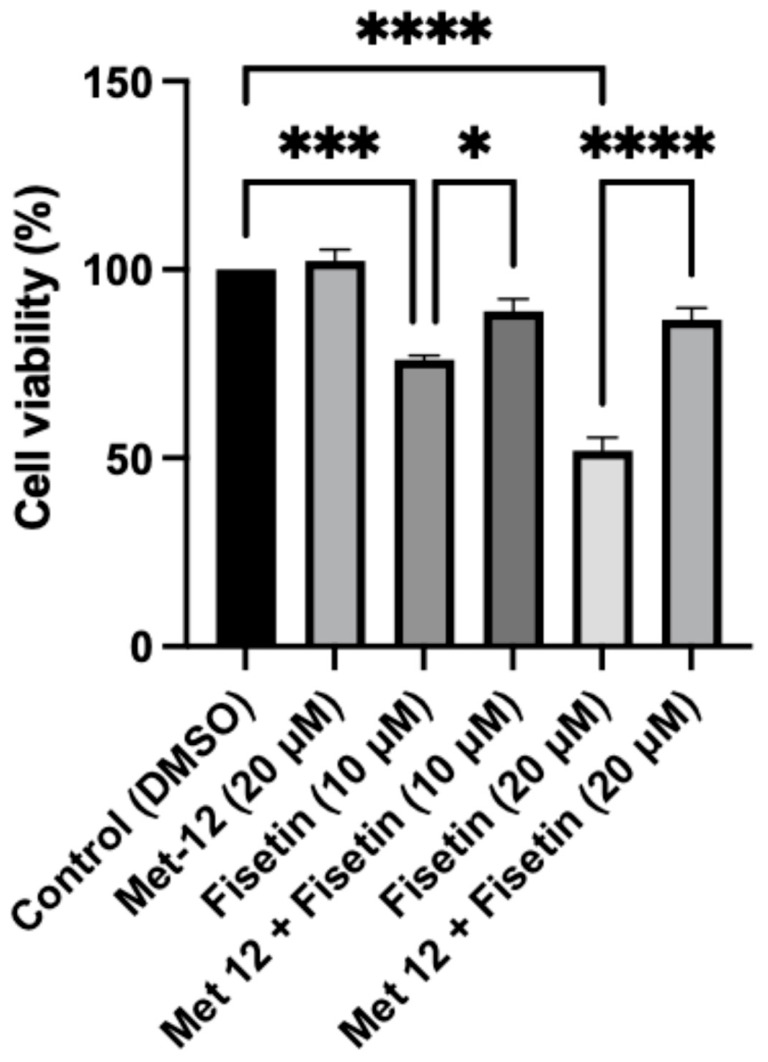
The effect of fisetin (10 and 20 µM) on Jurkat cell survival, measured using XTT assay. In the presence and absence of Fas receptor inhibitor Met-12. Data represented as mean ± SEM (*n* = 3) Statistical analysis carried out using ANOVA followed by Tukey’s *post hoc* test for significance (* *p* < 0.05) (*** *p* < 0.001) (**** *p* < 0.0001).

To assess whether fisetin modulated Fas receptor signalling, we conducted a toxicity study in Jurkat cells in the presence and absence of the Fas receptor inhibitor Met-12 (Figure 10). Fisetin (10µM) resulted in 76.1% viability, whereas pretreatment with Met-12 resulted in 89.0% viability (*p* = 0.0402). Furthermore, the higher concentration of fisetin (20 µM) resulted in 52.0% viability, which was rescued by Met-12 to 86.7% viability (*p* < 0.0001). It is also important to note that Met-12 did not affect cell viability *per se*. Pharmacological inhibition of Fas receptor signalling using Met-12 significantly attenuated fisetin-induced cytotoxicity, supporting the involvement of Fas-dependent signalling in mediating fisetin-induced leukaemic cell death.

### 2.8. Synergistic Effects of Fisetin in Combination with Pinocembrin in Jurkat and K562 Cells

**Figure 11:**
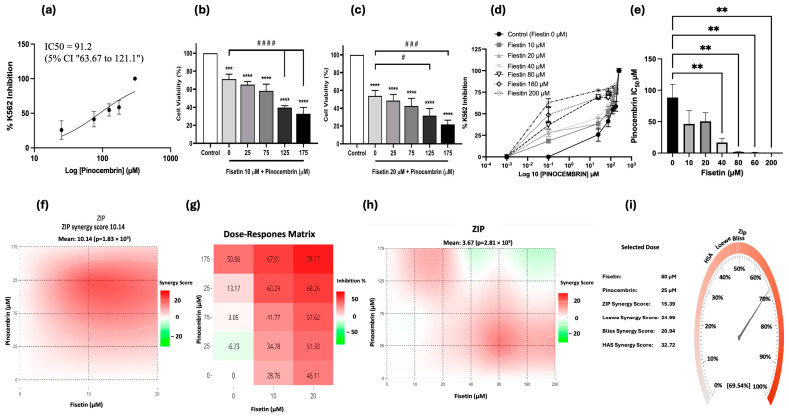
The synergistic cytotoxic effects of fisetin and pinocembrin in K562 and Jurkat cells. (**a**) % Cell viability of Jurkat cells treated with varying concentrations of pinocembrin + 10 µM fisetin. (**b**) % Cell viability of Jurkat cells treated with varying concentrations of pinocembrin + 20 µM fisetin. (**c**) Dose–response of pinocembrin treatment of K562 cells in the absence of fisetin. (**d**) Dose–response for pinocembrin in the presence of fisetin (0–200 µM). (**e**) IC50 of pinocembrin in the presence of varying concentrations of fisetin. (**f**) Synergistic interaction analysis of fisetin and pinocembrin using the ZIP model, showing an overall synergy score of 10.14 in Jurkat cells. (**g**) Dose–response matrix illustrating the percentage of inhibition across different concentration combinations, where red indicates higher inhibition and green indicates lower inhibition in Jurkat cells. (**h**) Synergistic interaction analysis of fisetin and pinocembrin using the ZIP model, showing an overall synergy score of 3.67 in K562 cells. (**i**) Synergy assessment of combination of fisetin and pinocembrin at the selected concentration. The selected dose concentration of fisetin and pinocembrin exhibited high-synergy interaction as indicated by positive (red) ZIP, Loewe, Bliss and HSA synergy scores suggesting enhanced anti-leukaemic effect of the combined treatment at comparatively low concentration compared to the individual treatments. Statistical significance was analysed using one-way ANOVA followed by Tukey’s post hoc test compared with the control DMSO (** *p* < 0.01, *** *p* < 0.001, **** *p* < 0.0001) and compared with fisetin alone (# *p* < 0.05, ### *p* < 0.001, #### *p* < 0.0001).

Based on the previously determined effects of fisetin and pinocembrin alone, the viability of Jurkat cells was assessed using the XTT assay. The half-maximal inhibitory concentration (IC_50_) for fisetin was 19.23 ± 1.14 µM (Figure 1f), while that of pinocembrin was 174.97 ± 1.25 µM. Therefore, the cytotoxic effects of the combination were evaluated using concentrations below IC_50_ values. Jurkat cells were treated with fisetin (10 and 20 µM) in combination with pinocembrin (25, 75, 125 and 175 µM) for 48 h. As shown in Figure 11a, treatment with 10 µM fisetin, in combination with increasing concentrations of pinocembrin (25 to 175 µM), resulted in a significant, concentration-dependent decrease in cell viability compared to the control (DMSO < 1%). Similarly, co-treatment with 20 µM fisetin further enhanced the cytotoxic effect (Figure 11b). Specifically, combinations with higher pinocembrin concentrations (125 and 175 µM) led to a significant reduction in cell viability compared with fisetin alone (Figure 11b). For K562, the half-maximal inhibitory concentration (IC_50_) for fisetin was 193.7 µM (Figure 1g), and we evaluated the half-maximal inhibitory concentration (IC_50_) of pinocembrin using the XTT assay after treating cells with pinocembrin (25, 75, 125, and 175 µM) for 48 h, which came out to be 91.2 µM (Figure 11c). To assess the combination’s cytotoxic effect, K562 cells were treated with fisetin (10, 20, 40, 80, 160, and 200 µM) in combination with pinocembrin (25, 75, 125, and 175 µM) for 48 h. The dose–response curve demonstrated that increasing concentrations of fisetin (10 to 200 µM) in combination with increasing concentrations of pinocembrin (25 to 175 µM) resulted in significant, concentration-dependent growth inhibition compared to the control (DMSO < 1% in K562 cells) (Figure 11d). We further evaluated the IC_50_ values of pinocembrin in the presence of increasing concentrations of fisetin (10, 20, 40, 80, 160, and 200 µM) in K562 cells. Pinocembrin alone exhibited the IC_50_ value of 91.2 µM, as shown in Figure 9c, while co-treatment with fisetin led to a progressive reduction in the IC_50_ value of pinocembrin, with a significant decrease seen at 40 µM of fisetin, and near-complete sensitisation was observed at higher fisetin concentrations (80–200 µM) (Figure 11e). Interestingly, these findings suggest a potential synergistic interaction between fisetin and pinocembrin and enhanced sensitivity to leukaemic cells, particularly at concentrations below their IC_50_ values.

To evaluate the synergistic effects of fisetin and pinocembrin, the viability data were analysed using Synergy Finder Plus software (version 07.09.2024-R-3.10.3). The interaction between two compounds is quantified as a ZIP synergy score, which can be interpreted as follows: scores below −10 indicate antagonism, values between −10 and 10 indicate an additive effect, and scores above 10 indicate synergism.

The overall ZIP synergy score for the fisetin–pinocembrin combination in Jurkat cells was 10.14 (Figure 11f). The most synergistic area was observed at 125 µM pinocembrin combined with 10 µM fisetin (ZIP score = 20.01), followed by 125 µM pinocembrin with 20 µM fisetin (ZIP score = 14.52). Moderate synergy was also detected at 75 µM pinocembrin in combination with fisetin (ZIP scores ranging from 11.82 to 14.27). The inhibitory dose–response matrix also showed greater inhibition at higher pinocembrin concentrations when combined with fisetin (Figure 11g). We also calculated the overall synergy of these drugs in K562 cells using the same model for ZIP synergy score, and the ZIP synergy score for the fisetin and pinocembrin combination in K562 cells was 3.67 (Figure 11h), which was comparatively low compared to Jurkat cells. However, the most synergistic area was observed at 25 µM pinocembrin combined with 80 µM fisetin (Figure 11i) (ZIP score = 15.39), indicating synergy at specific concentrations which are very low when compared to their individual IC_50_ values, indicating high synergism.

### 2.9. Effects of Fisetin and Pinocembrin on Clonogenic Survival in Leukaemic Cells

**Figure 12:**
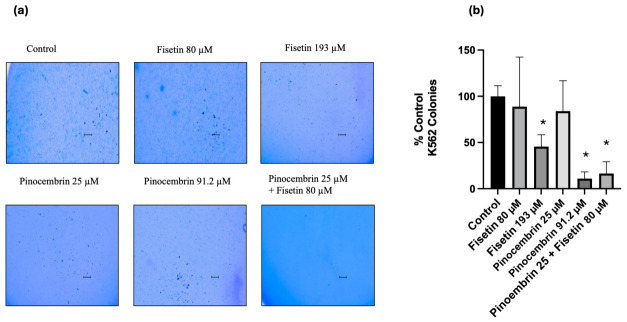
Effects of fisetin and pinocembrin on clonogenic survival of K562 leukaemic cells. (**a**) Colonies of over 50 cells formed by K562 cells in soft agar following treatment with fisetin, pinocembrin and their combination. Control cells formed several well-defined colonies, whereas treatment with fisetin at its IC_50_ (193.7 µM) or pinocembrin at its IC_50_ (91.2 µM) significantly reduced colony formation. Combination treatment with fisetin (80 µM) and pinocembrin (25 µM) markedly suppressed clonogenic growth compared to control and single-drug treatments, whereas no significant reduction was seen when treated alone at these concentrations. (**b**) Quantitative analysis of colony numbers following different treatments. Data are presented as mean ± SEM from three independent experiments (*n* = 3). Statistical significance was determined compared to control and/or indicated treatment groups (* *p* < 0.05).

To evaluate the long-term effects of fisetin and pinocembrin on leukaemic cell survival, a soft-agar colony formation assay was performed in K562 cells. Numerous well- defined colonies were observed in control cells, indicating robust clonogenic potential (Figure 12a,b). Treatment with fisetin and pinocembrin at their IC_50_ concentrations (193.7 µM and 91.2 µM, respectively) significantly reduced colony numbers compared with the control group, indicating decreased clonogenic potential. Cells exposed to pinocembrin at 25 µM and fisetin at 80 µM also did not show a significant reduction in colony number, consistent with their effects observed in short-term viability assays. Importantly, the combination of fisetin (80 µM) and pinocembrin (25 µM), identified as a synergistic condition in viability assays, led to a pronounced suppression of clonogenic growth, as evidenced by a marked reduction in colony number relative to the control and single-drug treatments (Figure 12a,b). These findings demonstrate that co-treatment with pinocembrin enhances the cytotoxic and clonogenic suppressive effect of fisetin, supporting a synergistic interaction that extends beyond acute cytotoxicity to sustained suppression of colony-forming ability. We also assessed clonogenic survival in Jurkat cells under similar conditions; however, these cells exhibited poor colony-forming efficiency and failed to generate well-defined colonies in soft agar. This observation is consistent with previous reports indicating that Jurkat cells have limited clonogenic potential in semi-solid media [34].

### 2.10. Effects of the Combination of Fisetin and Pinocembrin on Protein Expression

**Figure 13:**
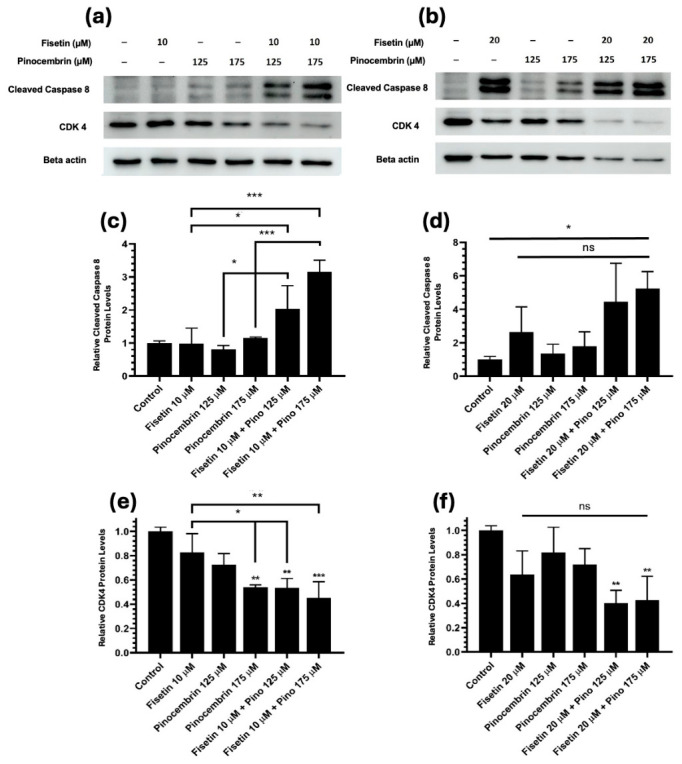
Effect of the combination of fisetin and pinocembrin (Pino) on protein expression in Jurkat cells. Jurkat cells were treated with fisetin at 10 µM (**a**) and 20 µM (**b**) in combination with pinocembrin (125 and 175 µM) for 48 h. (**c**–**f**) Protein expression levels were determined by Western blot analysis. (**c**–**f**) Relative protein expression levels are presented as the mean ± SEM from three independent experiments (*n* = 3). Statistical significance was analysed using one-way ANOVA followed by Tukey’s *post hoc* test (* *p* < 0.05, ** *p* < 0.01, *** *p* < 0.001, ns = not significant).

After establishing that fisetin and pinocembrin cause cell death, subsequent mechanistic studies focused primarily on fisetin concentrations of 10–20 µM, which encompass the IC_50_ value in Jurkat cells and therefore represent the most biologically relevant concentration range for investigating apoptotic signalling. The effects of fisetin and pinocembrin, both individually and combined, on the expression of apoptotic and cell cycle-related proteins in Jurkat cells were analysed using Western blot following a 48 h treatment with fisetin (10 and 20 µM) alongside pinocembrin (125 and 175 µM), as illustrated in Figure 13a–f. In Figure 13a,c, treatment with fisetin at 10 µM or pinocembrin (125 and 175 µM) alone caused only slight, statistically insignificant changes in cleaved caspase-8 protein expression.

However, combined treatment with fisetin and pinocembrin significantly increased cleaved caspase-8 levels compared with treatment with either compound alone. Notably, fisetin at 10 µM combined with pinocembrin at 125 or 175 µM showed higher expression than either compound alone. Previously, fisetin markedly increased cleaved caspase-8 protein expression in a concentration-dependent manner, particularly at higher concentrations (20 µM), as demonstrated in Figure 6b. 

Likewise, Figure 13d shows elevated cleaved caspase-8 protein expression with fisetin at 20 µM alone; however, the combination groups with fisetin at 20 µM did not show statistically significant differences. As reported in a previous study, pinocembrin induces G0/G1 cell cycle arrest by inhibiting CDK4 (36), thereby preventing entry into S phase and suppressing cell proliferation in Jurkat cells. As shown in Figure 13e–f, CDK4 protein expression was reduced following the combination treatment. In particular, combined treatment with fisetin at 10 µM and pinocembrin (125 and 175 µM) resulted in a significant downregulation of CDK4 protein levels compared with fisetin or pinocembrin alone. In contrast, single treatments caused slight decreases that were not statistically significant. Collectively, these findings indicate that the combination of fisetin and pinocembrin promotes the extrinsic apoptotic pathway, as evidenced by increased cleaved caspase-8 levels, and inhibits cell cycle progression by downregulating CDK4 in Jurkat cells.

## 3. Discussion

This is the first study to show that fisetin exhibited significant anti-leukaemic activity across different haematological malignancies, including lymphoid (Jurkat) and myeloid (K562) leukaemia cells. While previous studies have demonstrated anticancer activity of fisetin in a variety of solid and haematological malignancies, the present study extends these observations by demonstrating differential sensitivity between lymphoid and myeloid leukaemia cells, identifying synergistic interactions between fisetin and pinocembrin, and providing functional evidence supporting the involvement of Fas-dependent apoptotic signalling through pharmacological inhibition studies. These findings contribute new mechanistic and therapeutic insights into the potential application of flavonoid-based combination strategies in leukaemia.

A differential sensitivity range was observed between the two cell types, where Jurkat cells were more responsive to fisetin treatment, whereas K562 cells required higher concentrations to achieve comparable cytotoxic effects, highlighting cell line-specific differences in drug responsiveness, which can be attributed to the differences in cellular origin, distinct signalling pathways, and apoptotic susceptibility between lymphoid and myeloid leukaemia cells.

An important consideration is the relatively high concentration of fisetin required to induce cytotoxicity, particularly in K562 cells. Fisetin exhibits limited aqueous solubility and relatively low oral bioavailability, which may restrict achievable systemic exposure following dietary consumption, but can be improved with pharmaco-enginering [35]. Nevertheless, fisetin has demonstrated anticancer activity in numerous preclinical studies at similar concentration ranges, and several formulation strategies, including nanoformulations and encapsulation approaches, have been developed to improve bioavailability and tissue exposure. Furthermore, the enhanced activity observed following combination with pinocembrin suggests that combination-based approaches may reduce the concentration requirements of individual compounds. Future pharmacokinetic and in vivo studies will be necessary to establish the clinical relevance of these findings.

Similarly, previous studies have shown that fisetin enhances cytotoxicity in human chronic B-cell leukaemia cell lines (EHEB and HG-3) [36]. It decreases the percentage of viable cells, with IC_50_ values of 50 ± 2 μM in HG-3 cells and 38 ± 2 μM in EHEB cells. The findings also indicate that fisetin primarily induces apoptotic cell death rather than classical cell cycle arrest in leukaemic T cells, as indicated by increasing the activities of caspase-3 and caspase-9.

We have also shown that there is very little toxicity in HaCaT and HEK cells. However, these immortalised non-haematopoietic cell lines may not fully recapitulate primary non-malignant physiology. The observed selectivity therefore likely reflects lineage- and context-dependent differences in apoptotic sensitivity rather than a strict cancer-versus-normal distinction. Furthermore, previous studies with fisetin have shown no cytotoxic effects on PBMCs obtained from healthy volunteers, even at concentrations up to 100 μM [36].

In agreement with previous reports, fisetin induces cytotoxicity in acute promyelocytic leukaemia (HL60) cells, leading to cell death, with an IC_50_ of approximately 30 μM at 24 h, as determined by the MTT assay. Interestingly, fisetin did not affect the viability of normal human lymphocytes, even at concentrations up to 200 μM [37]. This study indicates that fisetin inhibits cell proliferation without affecting normal cells, an important property for the design of safer anticancer agents. Mechanistically, this study showed that fisetin predominantly activates the extrinsic apoptotic pathway. It did not cause cell cycle arrest in the G0/G1, S, and G2/M phases at 24 and 48 h in Jurkat cells, compared with the control (DMSO < 1%).

However, flow cytometry revealed a concentration-dependent increase in the sub-G1 population, indicating enhanced apoptosis. This was further supported by Annexin V/PI staining, which showed a significant, concentration-dependent increase in apoptotic cells at 10, 20, 40, and 80 µM fisetin. These observations are consistent with previous reports demonstrating that fisetin promotes apoptosis, as indicated by increased Annexin V^+^/PI^−^ and Annexin V^+^/PI^+^ cell populations in various cancer cell types, including breast cancer (4T1 cells) [38], glioblastoma (U-138 MG cells) [39], gastric carcinoma (AGS cells) [40], cervical cancer (HeLa cells) [41], pancreatic cancer (PANC-1 cells) [42] and acute monocytic leukaemia (THP-1 cells) [43]. Notably, these findings demonstrate that fisetin also effectively induces apoptosis in human leukaemic T cells (Jurkat cells), supporting its potential as a promising anticancer agent.

Apoptosis is regulated through two main signalling pathways: the intrinsic (mitochondrial) pathway and the extrinsic (death receptor) pathway. Intracellular stress signals activate the intrinsic pathway and involve the regulation of Bcl-2 family proteins, including Bax and Bcl-2, as well as the release of cytochrome c from the mitochondria. Conversely, the extrinsic pathway is initiated when extracellular ligands bind to death receptors, such as Fas and tumour necrosis factor receptors (TNFRs), thereby activating caspase-8. Both pathways converge on caspase-3 activation, resulting in DNA fragmentation, apoptotic cell death and phagocytic clearance [19,20]. The molecular mechanistic studies suggest that fisetin predominantly activates the extrinsic apoptotic pathway in Jurkat cells. Increased activation of cleaved caspase-8 protein and absence of significant cleaved caspase-9 activation and procaspase-9 protein expression suggests absence of dominant mitochondrial apoptotic signalling under the present experimental conditions.

Fisetin treatment increased Bcl-2 expression while Bax expression showed a slight, non-significant decrease, resulting in a reduced Bax/Bcl-2 ratio. These findings further support that fisetin does not effectively activate the mitochondrial (intrinsic) apoptotic pathway under these conditions. Instead, fisetin induces apoptosis in Jurkat cells potentially through activation of the extrinsic apoptotic pathway, as indicated by increased caspase-8 activation. Previous studies have reported that fisetin induces apoptosis in human cervical cancer HeLa cells through ERK1/2-mediated activation of caspases-3 and -8 and the cleavage of poly (ADP-ribose) polymerase [41]. Moreover, fisetin induced apoptotic cell death in human thyroid TPC-1 cancer cells, as evidenced by decreased cell viability, disruption of mitochondrial membrane potential (MMP) and alterations in cell cycle phases. This effect was associated with increased expression of caspase-3, -8, and -9, along with suppression of the JAK/STAT3 signalling pathway [44]. Similarly, fisetin induces apoptosis in human colon cancer HCT-116 cells via the activation of both extrinsic (death receptor) and intrinsic (mitochondrial) pathways. This is evidenced by increased protein levels of cleaved caspase-8, Fas ligand (Fas-L), death receptor 5 (DR5) and TNF-related apoptosis-inducing ligand (TRAIL), as well as caspase activation. In addition, fisetin promotes PARP cleavage, mitochondrial membrane disruption and cytochrome c release [45]. These findings in other cancer cell types are consistent with those of the present study, indicating that fisetin can induce apoptosis in human leukaemic T cells via the extrinsic apoptotic pathway. The observed increase in Bcl-2 expression may reflect alternative mechanisms; such findings are frequently reported in drug-resistant cancers and could be linked to tumour progression or enhanced cellular survival, particularly through the regulation of autophagy [33]. Therefore, it is plausible that during the early stages of survival mechanism activation, the treated cells undergo anti-apoptotic processes, resulting in increased Bcl-2 expression (an anti-apoptotic protein).

Interestingly, fisetin increased Bcl-2 expression despite inducing apoptosis. Similar paradoxical increases in Bcl-2 have been reported in stressed cancer cells and may represent a compensatory pro-survival response. In addition to its anti-apoptotic function, Bcl-2 participates in the regulation of autophagy through interactions with Beclin-1, suggesting that the observed increase may reflect broader cellular stress adaptation mechanisms.

Macroautophagy (autophagy) and apoptosis are cellular processes that regulate survival and death, and their interplay varies across biological contexts [17,18]. Previous studies have reported that increased Bcl-2 expression suppresses autophagy by interacting with the protein Beclin-1 and preventing its involvement in initiating autophagosome formation [46]. Autophagy involves the sequestration of cytoplasmic components into autophagic vacuoles for lysosomal degradation. Upon induction, a phagophore forms through Beclin-1-mediated nucleation, elongates, and engulfs cytosolic material to generate an autophagosome. This process is regulated by autophagy-related (ATG) proteins and includes nucleation, elongation, and completion. Autophagosomes then fuse with lysosomes to form autolysosomes, where cargo is degraded, and metabolites are released into the cytosol, followed by lysosome reformation through autophagic lysosome reformation (ALR) [47,48,49]. Whereas LC3-II (derived from LC3-I) serves as a key marker of autophagosomes, selective cargo is recruited via receptors such as SQSTM1/p62. SQSTM1/p62 links ubiquitinated cargo to autophagosomes and is degraded during autophagy; therefore, its accumulation generally indicates impaired or reduced autophagic flux, the process involving autophagosome–lysosome fusion and degradation [50,51].

In this study, fisetin significantly increased SQSTM1/p62 expression in a concentration-dependent manner, whereas LC3B protein levels did not change significantly. The accumulation of p62 suggests that fisetin alters autophagy-related signalling and may affect autophagic homeostasis.

However, because LC3B levels remained largely unchanged and chloroquine-based flux analysis did not provide definitive evidence of complete autophagic blockade, the precise effect of fisetin on autophagic flux remains unclear. Increased BCL-2 expression observed following fisetin treatment may further contribute to modulation of autophagy-related pathways. Collectively, these findings suggest that fisetin influences autophagy-associated signalling, although additional studies employing dedicated autophagic flux assays will be required to fully define the underlying mechanism.

Previous studies have shown that fisetin suppresses autophagy and induces apoptosis in colorectal cancer cells (SW-480 cells). This is evidenced by fluorescent autophagic vacuoles (AVOs), decreased autophagy markers (Beclin-1 and LC3), p62 accumulation, increased Annexin V/propidium iodide (+/+)-positive cells, elevated cleaved caspase-3 and nuclear PARP-1, mitochondrial membrane potential collapse, cytochrome c release, and Nrf2 suppression [52].

To further support the finding of this study that fisetin activates the extrinsic apoptotic pathway, which occurs when extracellular ligands bind to death receptors on the cell surface, molecular docking analysis was performed using fisetin as the ligand and the Fas receptor as the target. The docking analysis suggested the potential for interaction between fisetin and Fas, with a calculated binding free energy (ΔG) of −7.0274 kcal/mol, suggesting that fisetin forms a stable and energetically favourable complex with the Fas receptor. Fisetin potentially interacts with the Fas receptor through multiple non-covalent interactions. Although docking analysis alone cannot confirm direct receptor activation, these findings, along with the robust activation of caspase-8, further support our hypothesis that fisetin may contribute to death receptor-mediated apoptotic signalling.

The molecular docking analysis (intended as hypothesis-generating rather than definitive evidence of direct receptor binding) predicts a favourable interaction between fisetin and the Fas receptor; however, docking studies alone cannot establish direct receptor binding. Consequently, the docking results should be considered supportive computational evidence. Additional biophysical approaches, such as molecular dynamics simulations or receptor-binding studies, will be required to further characterise the interaction.

Subsequent mechanistic validation using the Fas antagonist Met-12 was performed at 10–20 µM fisetin, supporting the biological relevance of this concentration range. The modulation of Fas signalling is strongly suggested by Met-12 recovery of cell survival in Jurkat cells treated with fisetin. These data strongly suggest that fisetin modulates Fas signalling, although whether this occurs through direct interaction, as suggested by the docking study, or through other mechanisms remains to be elucidated.

Previous studies have shown that physalin A binds to the Fas-FADD death receptor complex, forming a stable complex via hydrogen bonds and hydrophobic interactions in molecular docking analyses, thereby inducing apoptosis and inhibiting cell survival in oral squamous carcinoma [53]. Moreover, the extracellular ligands binding to death receptors, such as the Fas receptor, which contains multiple extracellular cysteine-rich domains (CRDs) primarily involved in ligand binding, lead to the recruitment of the Fas-associated death domain (FADD) and the activation of caspase-8 via the death-inducing signalling complex (DISC) [54,55,56]. Therefore, fisetin may interact with the Fas receptor and modulate Fas-mediated apoptotic signalling, consistent with previous experimental results indicating that fisetin activates the extrinsic apoptotic pathway.

This study is the first to demonstrate that the combination of fisetin and pinocembrin significantly enhanced cytotoxicity, reducing cell viability compared with fisetin alone in Jurkat and K562 cells. The combination exhibited stronger and more broadly distributed synergistic interactions in Jurkat cells, whereas K562 cells demonstrated more modest synergy that was restricted to specific concentration combinations. These findings suggest that the response to fisetin–pinocembrin combination therapy may be influenced by leukaemia subtype and cellular context. Importantly, fisetin and pinocembrin are chemically defined small molecules rather than complex biological extracts, supporting reproducibility and facilitating their evaluation as mechanistically targeted therapeutic agents. The enhanced inhibitory effect observed at sub-IC_50_ concentrations suggests that combination treatment may improve therapeutic efficacy while minimising dose-dependent toxicity. Combination therapies are quite attractive in leukaemia, as they reduce the risk of systemic toxicity and resistance. In this study, synergy analysis demonstrated that fisetin and pinocembrin produced synergistic interactions in leukaemic cells at comparatively lower doses, supporting the possibility that these compounds act through complementary molecular mechanisms to enhance anticancer efficacy [57].

Similarly, previous studies have shown that fisetin is a potential acetylcholinesterase (AChE) inhibitor implicated in Alzheimer’s disease and may serve as an adjunct to galantamine. The combination of fisetin (2.25 μM) and galantamine (0.2 μM) exhibited a remarkable synergistic effect, with a ZIP synergy score of 13.47 from the online Synergy Finder Plus software (version 07.09.2024-R-3.10.3) [58]. Several studies have reported synergistic anticancer effects of fisetin when combined with other bioactive natural compounds and conventional chemotherapy [59]. Such effects have been observed in various cancer cell types, including ovarian cancer (A2780 and OVCAR-3 cells) [60,61], cervical cancer (HeLa cells) [62], colon cancer (COLO205 cells) [63], colorectal cancer (HT29 cells) [64] and breast cancer (MCF7 and MDA-MB-231 cells) [65]. However, fewer studies have reported on leukaemia.

Importantly, the effects of fisetin and pinocembrin combination extended beyond short-term cytotoxicity, as demonstrated by the colony formation assay in K562 cells. The combination treatment significantly suppressed clonogenic survival in K562 cells, thereby reducing colony numbers. This indicates that the combination not only induces acute cell death but also deregulates the long-term proliferative capacity of leukaemic cells. These assays were not feasible in Jurkat cells due to their inherently low colony-forming efficiency in semi-solid media, which is consistent with previous observations for certain T-cell leukaemia lines [34]. So, in this study, we demonstrate that the combination of fisetin and pinocembrin significantly enhances cytotoxicity and exhibits a synergistic effect in human leukaemic cells, supporting its potential as an anticancer agent. This approach can achieve effective treatment outcomes and may help reduce the required chemotherapy dosage, thereby reducing adverse effects.

Fisetin has the ability to promote caspase-8-dependent extrinsic apoptosis that can be advantageous in resistant leukaemia conditions and evade mitochondrial apoptosis-based therapies. Importantly, significant enhancement in fisetin-mediated apoptotic signalling and cytotoxicity at sub-IC_50_ concentrations when co-treated with pinocembrin suggests that mechanistically complementary combinations may improve therapeutic responses while potentially reducing toxicity associated with conventional chemotherapy. Similarly, previous studies have shown that the combination of fisetin and sorafenib, an oral targeted therapy, synergistically induced apoptosis in cervical cancer (HeLa cells), accompanied by a marked loss of mitochondrial membrane potential (MMP). This effect was mediated by activation of caspase-3 and caspase-8, increased Bax/Bcl-2 ratio, and subsequent PARP cleavage [62]. Moreover, the combination of doxorubicin (DOX), a chemotherapeutic agent, and fisetin significantly reduced cell viability and induced apoptosis, as evidenced by membrane blebbing and chromatin condensation. This synergistic effect enhances apoptosis in lymphoma (DL cells) via intracellular ROS generation, mitochondrial aggregation at the nuclear periphery, and upregulation of p53, Bax, cytochrome c, caspase-3, and cleaved caspase-9 [66].

Collectively, our data support a model in which fisetin promotes extrinsic apoptosis through Fas-associated signalling, as indicated by molecular docking, Fas inhibition and caspase-8 activation. Engagement of Fas is expected to drive formation of the death-inducing signalling complex (DISC), facilitating procaspase-8 activation and downstream apoptotic signalling, consistent with our observations. However, given that caspase-8 can also be activated via other death receptors, including TRAIL receptors and TNFR1, receptor-specific inhibition studies (e.g., Fas-blocking antibodies or siRNA) and direct assessment of DISC assembly will be required in future studies to definitively establish Fas specificity.

Collectively, these results suggest that combined treatment with fisetin and pinocembrin exerts synergistic anticancer effects by promoting apoptosis through activation of the extrinsic apoptosis pathway, as evidenced by upregulation of cleaved caspase-8, and by inhibiting cell cycle progression through downregulation of CDK4. Using both compounds together may allow lower concentrations of each agent while still achieving enhanced anticancer efficacy.

Therapeutic resistance and dose-limiting toxicity remain major barriers in the treatment of ALL, often driven by impaired apoptotic signalling, particularly within the intrinsic mitochondrial pathway. Therefore, targeting alternative mechanisms, such as death receptor-mediated apoptosis, represents a rational strategy to overcome resistance. In this study, molecular docking and downstream signalling data support a model in which fisetin affects Fas receptor signalling to activate caspase-8-dependent extrinsic apoptosis, thereby bypassing mitochondrial resistance pathways. Notably, co-treatment with pinocembrin significantly enhanced this effect, increasing apoptotic signalling and reducing cell viability at sub-IC_50_ concentrations. This synergistic interaction suggests that combining mechanistically complementary agents may improve therapeutic efficacy while enabling dose reduction, thereby limiting the toxicity associated with conventional chemotherapy. Collectively, these findings highlight the translational potential of targeting Fas-mediated apoptosis using defined small-molecule combinations in several leukaemia types.

Mechanistic conclusions were primarily derived from concentrations close to the IC_50_ value (10–20 μM), whereas higher concentrations used in Annexin V assays were included to define the maximal apoptotic response and may involve additional cellular targets. Collectively, in this study, fisetin, a chemically defined small molecule, is proposed to engage death receptor signalling, and potentially modulation of Fas receptor signalling, leading to recruitment of FADD and the possible formation of the death-inducing signalling complex (DISC). This would then promote activation of procaspase-8 and initiation of the extrinsic apoptotic cascade, as seen in our study. Downstream signalling results in apoptotic cell death, accompanied by inhibition of cell cycle progression via downregulation of CDK4. In parallel, fisetin treatment is associated with accumulation of p62, suggesting impaired autophagic signalling and further contributing to reduced cell survival. Co-treatment with pinocembrin enhances these effects, producing synergistic cytotoxicity and amplifying apoptotic signalling. While involvement of alternative death receptors cannot be excluded, this model highlights convergence on caspase-8 activation and supports a multi-targeted mechanism underlying the observed anti-leukaemic activity.

## 4. Materials and Methods

### 4.1. Chemicals

Fisetin (CAS No. 528-48-3) (catalogue number—HY-N0182) and pinocembrin (CAS No. 480-39-7) (catalogue number—HY-N0575) were both purchased from MedChemExpress (MCE) (Monmouth Junction, NJ, USA). Both compounds are well-characterised small molecules with defined chemical structures and known purity, enabling reproducible mechanistic investigation. Fisetin and pinocembrin were dissolved in dimethyl sulfoxide (DMSO) to prepare stock solutions at concentrations of 3 mM and 5 mM, respectively, and were stored at −20 °C until use. Concentrations were selected according to the biological endpoint under investigation. Sub-cytotoxic concentrations were used for cell cycle analysis to minimise confounding effects of extensive cell death, whereas concentrations spanning the IC_50_ value were used for mechanistic studies and apoptosis-related protein analysis. Higher concentrations were included in Annexin V assays to characterise the maximal apoptotic response.

### 4.2. Cell Cultures

The Jurkat T-cell line (ATCC) (Cellosaurus Accession# CVCL_0065) and the human chronic myelogenous leukaemia cell line K562 (ICell Bioscience Inc., Shanghai, China) (Cellosaurus Accession# CVCL_0004) were cultured under standard conditions in RPMI-1640 medium supplemented with L-glutamine and 10% foetal bovine serum (FBS) (Hyclone, GE Healthcare Life Sciences, Marlborough, MA, USA). HEK293 (ATCC) (Cellosaurus Accession# CVCL_0045) and HaCaT cells (ATCC) (Cellosaurus Accession# CVCL_0038) were cultured in DMEM, supplemented with 10% FBS and 1 × penicillin/streptomycin. Cells were incubated at 37 °C in a humidified atmosphere with 5% CO_2_.

### 4.3. Cell Viability Assay

The cytotoxic effects of fisetin and/or pinocembrin on cells grown in suspension, such as Jurkat and K562 cells, were evaluated using the trypan blue exclusion assay. Jurkat cells were seeded at a density of 1 × 10^5^ cells/mL and treated with various concentrations of fisetin (5 to 200 µM) for 48 h. After treatment, cells were stained with 0.4% trypan blue solution to determine the numbers of viable and nonviable cells. At the same time, the half-maximal inhibitory concentration (IC_50_) was determined using the XTT tetrazolium salt (Abcam, Cambridge, UK) with phenazine methosulfate (PMS) in both Jurkat and K562 cells. Both cells were seeded at a density of 1 × 10^5^ cells/mL and treated with various concentrations of fisetin (5 to 200 µM) for 48 h. For the XTT assay, XTT reagent was added, and the cells were incubated at 37 °C for 4 h. Afterwards, absorbance (Abs) was measured at 450 nm and 690 nm. Cell viability was calculated using the following equation: % Cell viability = (Abs of sample/Abs of control) × 100.

The cytotoxic effects of fisetin and/or pinocembrin in adherent cells such as HEK 293 or HaCaT cells were evaluated using the MTT assay. Cells were seeded in 96-well plates at an appropriate density (5 × 10^3^ to 1 × 10^4^ cells/well) and allowed to attach overnight under standard culture conditions (37 °C, 5% CO_2_).

After incubation, cells were treated with fisetin at concentrations of 10, 20, 40, 80, 160, and 200 µM, and pinocembrin at concentrations of 25, 75, 125, and 175 µM. For combination treatment studies, cells were treated with fisetin and pinocembrin combinations of 10 µM + 125 µM and 20 µM + 125 µM, respectively. Untreated cells served as the control group. Following 48 h treatment, 10 µL of MTT reagent (5 mg/mL in PBS) was added to each well and incubated for 3–4 h at 37 °C. After incubation, the medium containing MTT was carefully removed, and the resulting formazan crystals were dissolved in DMSO (100 µL/well). Absorbance was measured at 570 nm using a microplate reader.

### 4.4. Cell Cycle Assay

Cell cycle assay was performed using a propidium iodide (PI)/RNase staining solution (Invitrogen, Thermo Fisher Scientific, Waltham, MA, USA). Jurkat cells were seeded at a density of 1 × 10^5^ cells/mL and treated with fisetin at concentrations of 2.5, 5, 10 and 15 µM for 24 and 48 h. After that, the cells were collected, washed twice with cold 1X phosphate-buffered saline (PBS) (Hyclone, GE Healthcare Life Sciences, Chicago, IL, USA), and fixed in ice-cold 70% ethanol at −20 °C overnight. After fixation, cells were incubated with PI/RNase staining solution at room temperature in the dark for 15 min. The DNA content was subsequently analysed using a DxFLEX flow cytometer (Beckman Coulter, Pasadena, CA, USA).

### 4.5. Annexin V/PI Staining Assay

Apoptosis was assessed using an Annexin V-FITC/propidium iodide (PI) staining assay with a commercial detection kit (ImmunoTools, Friesoythe, Germany). Jurkat cells were seeded at a density of 1 × 10^5^ cells/mL and treated with fisetin at concentrations of 10, 20, 40 and 80 µM for 48 h. After treatment, cells were collected, washed twice with cold 1X phosphate-buffered saline (PBS), and resuspended in Annexin V binding buffer. The cell suspension was then incubated with Annexin V-FITC and PI at room temperature in the dark for 15 min. Apoptotic cell populations were immediately quantified using a BD FACSCelesta flow cytometer, and the resulting data were analysed with FACSDiva software version 07.09.2024-R-3.10.3.

### 4.6. Western Blot Analysis

Jurkat cells were seeded at a density of 1 × 10^5^ cells/mL and treated with fisetin at concentrations of 5, 10, 15 and 20 µM for 48 h. After treatment, cells were collected, washed twice with cold 1X phosphate-buffered saline (PBS), and lysed in RIPA buffer (Thermo Fisher Scientific, Waltham, MA, USA) supplemented with 1X protease inhibitor cocktail (Thermo Fisher Scientific, Rockford, IL, USA) on ice for 15 min. The total protein concentrations were quantified using a bicinchoninic acid (BCA) protein assay (Thermo Scientific, Waltham, MA, USA). Total protein at 10 µg was separated by 12% SDS-PAGE and transferred onto polyvinylidene difluoride (PVDF) membranes (Bio-Rad, Hercules, CA, USA). Membranes were blocked with 5% bovine serum albumin (BSA) in Tris-buffered saline containing 0.1% Tween-20 (TBST) for 1 h at room temperature, and then incubated overnight at 4 °C with primary antibodies. The following primary antibodies were tested: anti-CDK4 (1:3000, #12,790S), anti-p53 (1:1000, #2527S), anti-phosphorylated p53 (1:1000, #9284S), anti-cleaved caspase-8 (1:3000, #9496S), anti-procaspase-9 (1:1000, #9502S), anti-cleaved caspase-9 (1:1000, #9502S), anti-Bax (1:1000, #2774S), anti-Bcl-2 (1:3000, #4223S), anti-LCB3 (1:1000, #2775S), anti-SQSTM1/p62 (1:1000, #5114S) and anti-β-actin (1:10,000, #4970T) antibodies (Cell Signaling Technology, Danvers, MA, USA). Following incubation, membranes were washed three times with TBST and subsequently incubated with horseradish peroxidase (HRP)-conjugated anti-rabbit secondary antibodies (1:20,000, #7074S, Cell Signaling Technology, Danvers, MA, USA) for 2 h at room temperature. Protein bands were detected using an enhanced chemiluminescence (ECL) substrate (Bio-Rad, Hercules, CA, USA) and visualised with an Amersham ImageQuant 800 imaging system. Band intensities were quantified using ImageJ software version Fiji 1.0. the complete set of full uncropped western blots can be found in the Supplementary Materials (Supplementary Figures S1–S5).

### 4.7. Molecular Docking

The three-dimensional (3D) structure of fisetin was obtained from the PubChem database (PubChem CID: 5281614). The predicted structure of the Fas receptor, also known as CD95 or tumour necrosis factor receptor superfamily member 6 (TNFRSF6), was obtained from the AlphaFold Protein Structure Database (AlphaFold ID: AF-P25445-F1-v6). Molecular docking analysis was performed using the SwissDock web server (https://www.swissdock.ch/) (accessed on 15 December 2025) [67,68]. The docking results were assessed using predicted binding free energy values together with analysis of molecular interactions between fisetin and key amino acid residues of the Fas receptor. Docked complexes with the lowest (most negative) binding free energy (ΔG) values were regarded as the most energetically favourable ligand–receptor interactions. Three-dimensional molecular visualisations and molecular surface rendering were performed using UCSF Chimaera version 1.19 (build 42556). At the same time, a two-dimensional interaction diagram was analysed using BIOVIA Discovery Studio Visualizer version 25.1.0.24284 to provide detailed insight into ligand–protein interactions.

### 4.8. Met-12 Rescue of Fisetin Toxiticy in Jurkat Cells

Cells were treated with the Fas inhibitor Met-12 (CAS No. 1242964-46-0, purchased from MedChemExpress, catalogue number HY-P6440) at concentration of 20 µM and fisetin at concentrations of 10 µM and 20 µM, and combination treatments consisting of MET + fisetin (20 + 10 µM and 20 µM + 20 µM). Untreated cells served as the control group. Following 48 h treatment, XTT reagent was prepared according to the manufacturer’s instructions and added to each well. Plates were incubated for an additional 2–4 h at 37 °C until sufficient colour development was observed.

Absorbance was measured at 450 nm using a microplate reader and cell viability was calculated as a percentage relative to untreated control cells.

### 4.9. Cytotoxicity Assay of Combined Compounds and Synergy Analysis

Experiments were performed using different concentrations of each compound in a checkerboard layout to examine all possible pairwise combinations. This design enables a comprehensive evaluation of combination therapies. It supports the comparison of interaction effects using SynergyFinder Plus software (https://synergyfinder.org/) [57], which calculates synergy scores based on the zero interaction potency (ZIP) model to assess the effects of compound combinations.

To assess the cytotoxic effects of the combination, Jurkat cells were seeded at a density of 1 × 10^5^ cells/mL and treated with fisetin at 10 and 20 µM in combination with pinocembrin at 25, 75, 125, and 175 µM for 48 h. For K562 cells, fisetin (40, 80, 160, and 200 µM) was combined with pinocembrin (25, 75, 125, and 175 µM). After treatment, the cells were incubated with XTT reagent at 37 °C for 4 h. Absorbance was then measured at 450 nm and 690 nm. Cell viability was calculated using the following equation: % Cell viability = (Abs of sample/Abs of control) × 100. For combination studies, viability data were analysed using SynergyFinder Plus, which applies a bootstrapping method to generate surface response plots by comparing the observed data with a reference model represented by ZIP synergy scores.

### 4.10. Colony Formation Assay

The clonogenic inhibition potential of fisetin and pinocembrin in K562 cells was assessed using a soft-agar colony formation assay. Briefly, a base layer of 0.7% agar in complete RPMI-1640 medium was prepared and K562 cells were suspended in 0.3% agarose with complete medium at a density 5000 cells per well and overlaid onto the base layer. Cells were then treated with fisetin and pinocembrin either alone or in combination under the following conditions: control (DMSO < 1%), fisetin at its IC_50_ concentration, pinocembrin at its IC_50_ concentration, fisetin (80 µM), pinocembrin (25 µM), and a combination of fisetin (80 µM) and pinocembrin (25 µM) (a concentration where synergism was observed). Following treatment, cells were maintained for 10–14 days with periodic medium replacement. Colonies were subsequently fixed, stained with crystal violet, and quantified.

### 4.11. Statistical Analysis

The data are expressed as the mean ± standard error of the mean (SEM). Statistical differences were evaluated using one-way ANOVA, followed by Tukey’s or Dunnett’s multiple-comparison *post hoc* tests. Statistical significance was defined as *p* < 0.05.

## 5. Conclusions

In conclusion, this study demonstrated that fisetin inhibited Jurkat cell proliferation in a concentration-dependent manner, altered autophagic signalling, as evidenced by increased SQSTM1/p62 expression, and induced apoptosis via activation of the extrinsic apoptosis pathway, as indicated by Fas receptor signalling and increased cleaved caspase-8 protein expression. Moreover, molecular docking analysis suggested that fisetin may interact with the Fas receptor and activate Fas-mediated apoptotic signalling pathways. The combination of fisetin and pinocembrin enhanced cytotoxicity and showed synergistic effects in Jurkat cells (which may be particularly relevant in reducing toxicity associated with intensive ALL chemotherapy regimens) and K562 cells. Comparative analysis revealed differential sensitivity between leukaemia cell types, with Jurkat cells showing greater responsiveness than K562 cells, as reflected by their respective IC_50_ values. Importantly, the combination treatment significantly suppressed clonogenic survival in K562 cells, demonstrating inhibition of long-term proliferative capacity beyond acute cytotoxic effects. However, as bioactive flavonoids derived from natural sources, fisetin and pinocembrin are generally considered non-toxic [69]. Although the present study demonstrates anti-leukaemic activity in established leukaemia cell lines, future studies employing patient-derived leukaemia samples and in vivo leukaemia models will be necessary to validate the translational potential of fisetin and pinocembrin and to assess their efficacy within the complex tumour microenvironment. Further studies are also needed to elucidate the underlying mechanisms and assess the clinical potential of this combination in human leukaemic cells, with the aim of reducing chemotherapy dosage and associated adverse effects, and of developing novel therapeutic agents.

## Data Availability

Data is available from the corresponding author at reasonable request.

## Supplementary Materials

The following supporting information can be downloaded at https://www.mdpi.com/article/10.3390/ijms27125622/s1.

## Abbreviations

The following abbreviations are used in this manuscript:

AChE	acetylcholinesterase
ALR	autophagic lysosome reformation
ALL	acute lymphoblastic leukaemia
AMPK	AMP-activated protein kinase
APAF-1	apoptotic protease-activating factor 1
ARG	Arginine
ASP	aspartic acid
BCA	bicinchoninic acid
CAR	chimeric antigen receptor
CDKs	cyclin-dependent kinases
CR5	death receptor 5
CRD	cysteine-rich domains
CYS	Cysteine
Cyt c	cytochrome c
DISC	death-inducing signalling complex
DMSO	dimethyl sulfoxide
ECL	enhanced chemiluminescence
EMT	epithelial–mesenchymal transition
FADD	Fas-associated death domain
Fas-L	Fas ligand
FBS	foetal bovine serum
HRP	horseradish peroxidase
HSE	histidine
JAK	Janus kinase
LC3	microtubule-associated protein 1A/1B-light chain 3
LYS	lysine
MAPK	mitogen-activated protein kinase
MMP	mitochondrial membrane potential
PARP	poly ADP-ribose polymerase
PI	propidium iodide
PI3K	phosphoinositide 3-kinase
PMS	phenazine methosulfate
PVDF	polyvinylidene difluoride
ROS	reactive oxygen species
RPMI-1640	Roswell Park Memorial Institute 1640 medium
SER	serine
SQSTM1	sequestosome 1
STAT	signal transducer and activator of transcription
THR	threonine
TNFR	tumour necrosis factor receptors
ZIP	zero interaction potency
