# Supplementary material for: Synergistic Induction of Caspase-8-Mediated Leukaemic Cell Death by Fisetin and Pinocembrin
Source: Int J Mol Sci. 2026 Jun 22;27(12):5622. doi: 10.3390/ijms27125622 (PMC13300158; doi:10.3390/ijms27125622)

Supplementary Figure S1

Fisetin (uM) 24 hour

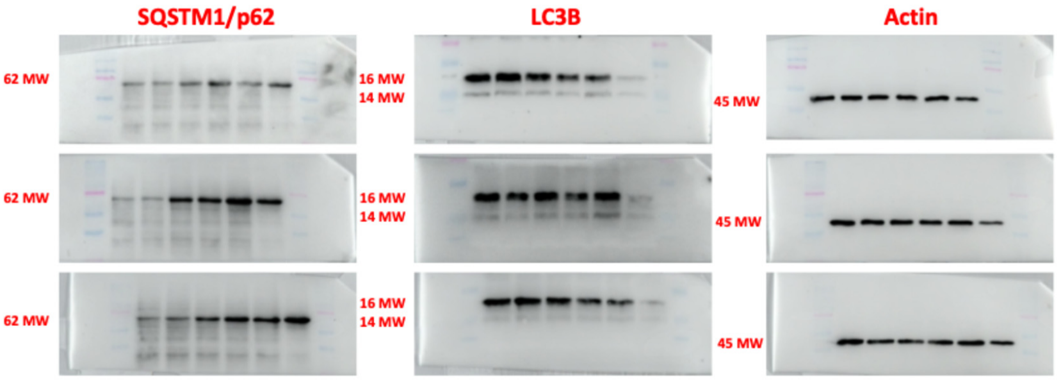

LCB3 1:1,000 (1:10,000)
SQSTM1/p62 1:1,000 (1:10,000)
Actin 1:10,000 (1:20,000)

Supplementary Figure S2

Fisetin 10 uM + Pinocembrin 48 hour

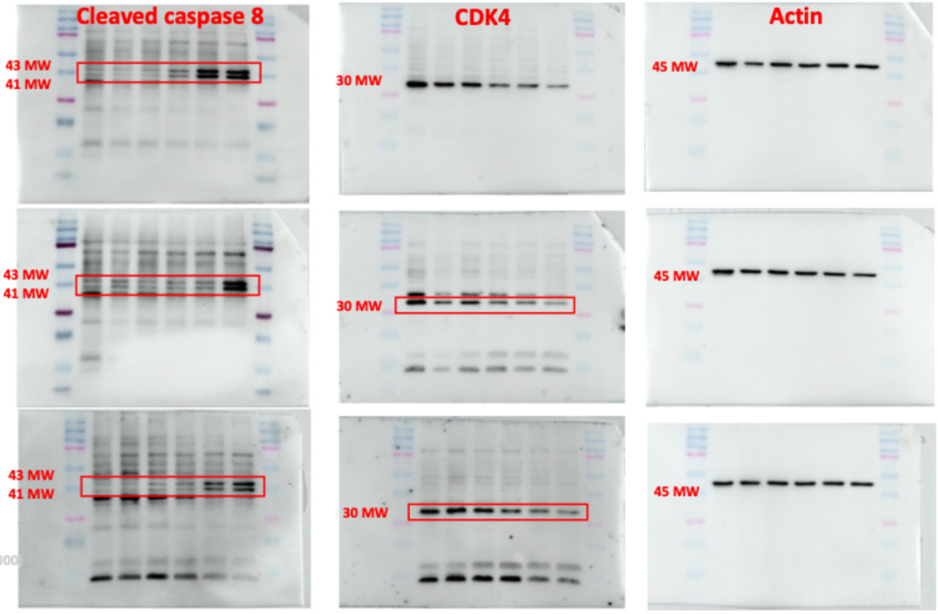

Cleaved caspase 8 1:3,000 (1:10,000)
CDK4 1:3,000 (1:10,000)
Actin 1:10,000 (1:20,000)

Supplementary Figure S3

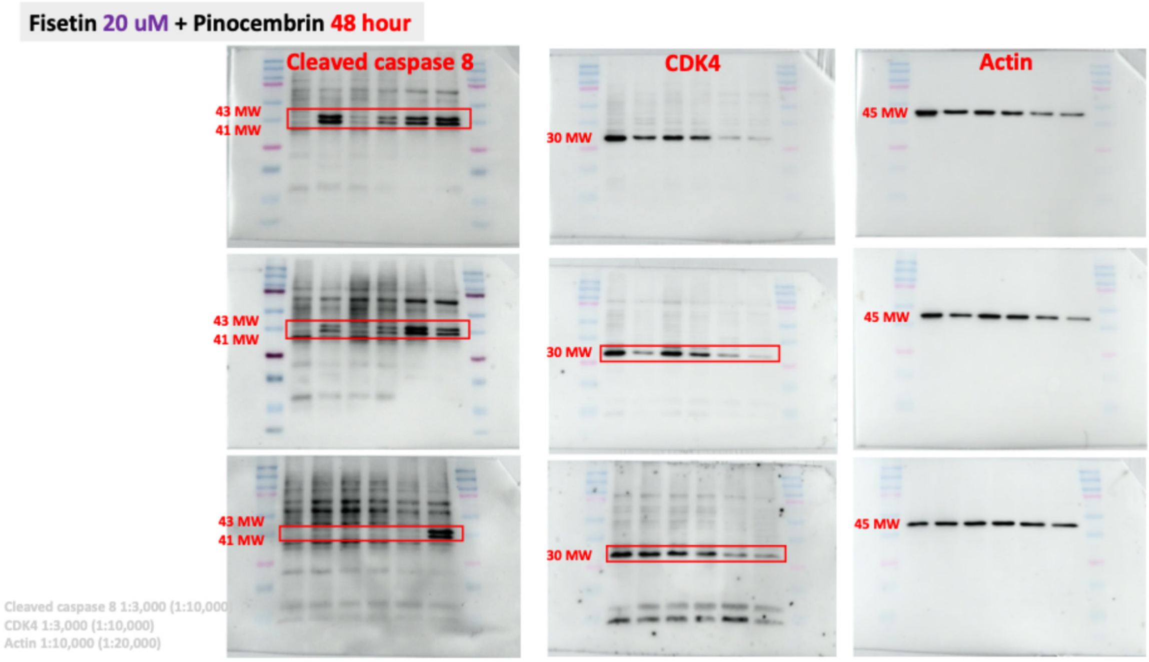

Supplementary Figure S4

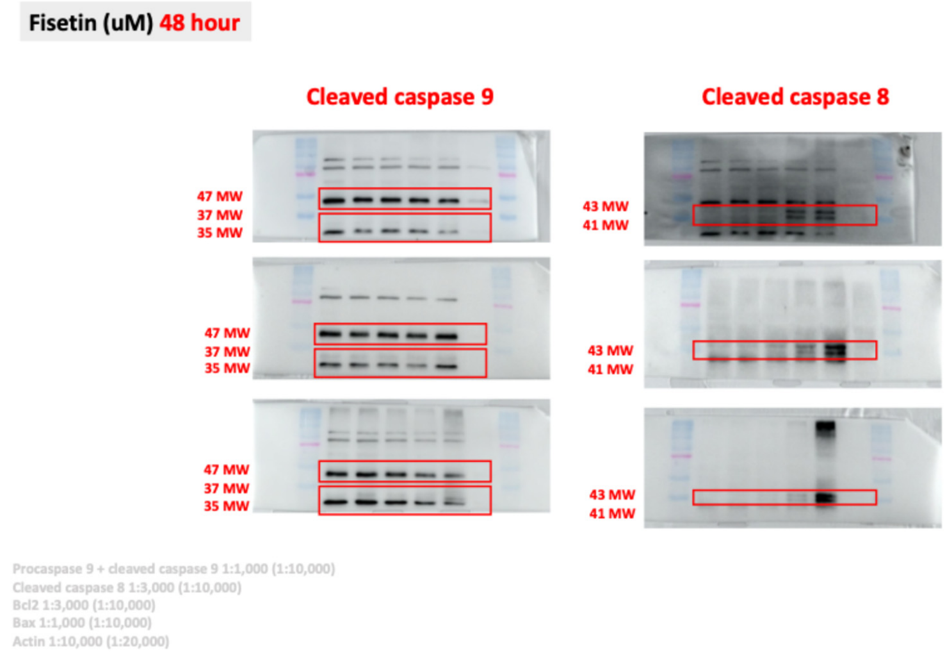

Supplementary Figure S5

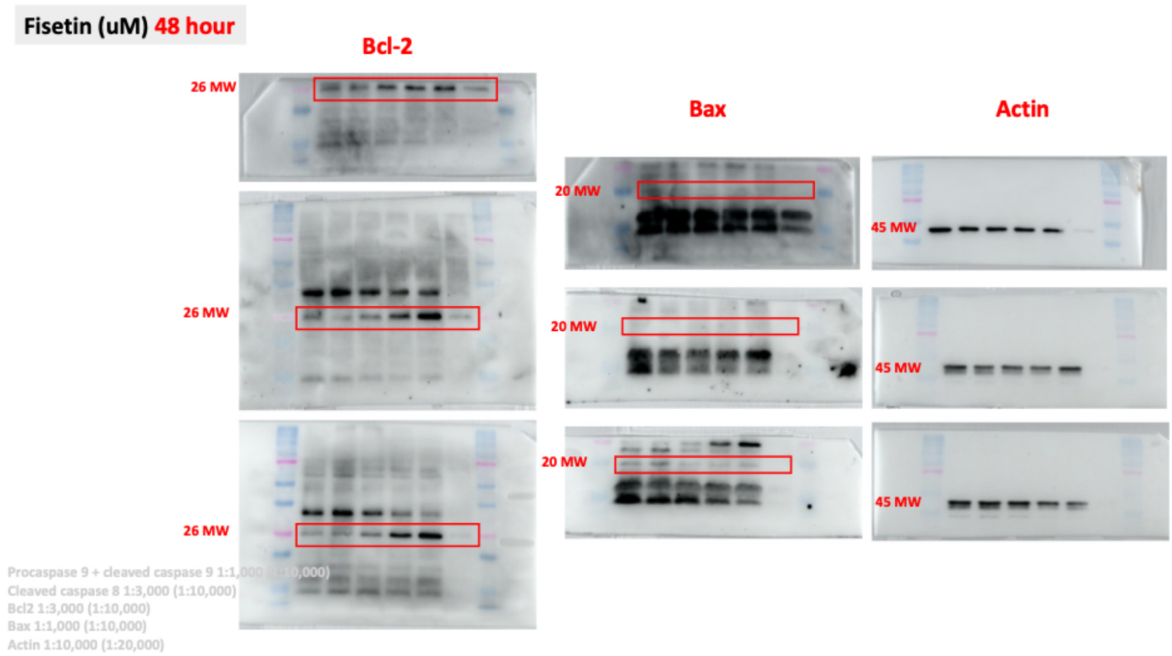

Supplement: Supplementary file 1 [file ijms-27-05622-s001.zip › ijms-4345262-supplementary.pdf]
